# An entropy based spatial–temporal cube with its application to assess stress of overburden due to mining

**DOI:** 10.1038/s41598-024-64453-2

**Published:** 2024-06-25

**Authors:** Changde Yang, Yang Chen, Binbin Yang, Chunshui Huang

**Affiliations:** 1 Key Laboratory of Xinjiang Coal Resources Green Mining, Ministry of Education, Xinjiang Institute of Engineering, Urumqi, 830023 China; 2 School of Mines, China University of Mining and Technology, Xuzhou, 221116 China; 3 School of Earth Sciences and Engineering, Sun Yat-sen University, Zhuhai, 519082 Guangdong China; 4 School of Civil Engineering, Xuchang University, No. 88 Bayi Rd, Xuchang, 461000 Henan China

**Keywords:** Fractal, Entropy, Rock stress, Similar material model, Civil engineering, Energy infrastructure

## Abstract

Underground coal seam mining significantly alters the stress and energy distribution within the overlying rock, leading to eventual structural degradation. Therefore, it is imperative to quantitatively identify the temporal and spatial characteristics of stress evolution of overlying rock caused by mining. This paper introduces a novel rock stress model integrating entropy and a spatial–temporal cube. Similar material model tests are used to identify the abrupt entropy changes within the mining rock, and the trend analysis is carried out to describe the spatial–temporal evolution law of stress during mining. Experimental findings indicate elevated stress levels in the unmined rock preceding and following the panel, as well as within specific rock strata above it. Definitively, dynamic stress arches within the surrounding rock of the stope predominantly bear and distribute the load and pressure from the overlying rock, and each stress mutation is accompanied by a sudden stress entropy change. Over time, z-score shows that the noticeable reduction in mining-induced overburden stress becomes increasingly pronounced, especially in the water-conducting fracture zone. The model's bifurcation set serves as the comprehensive criterion for the entropy-induced sudden changes in the rock system, signifying overall failure.

## Introduction

Rock mining failure is composed of rock engineering, groundwater system, underground mining environment and human activities, which has nonlinear, chaotic and self-organizing characteristics^1–5^. The nonlinear dynamic characteristics of rock during mining failure make it not only affected by traditional rock mechanics, but also affected by a variety of complex factors, thus forming a highly coupled nonlinear system^6–9^. Due to the non-uniformity of rock materials, various parameters of rock will also change, and the stress in rock will change at any time during mining^10–12^. Especially in the mine environment, because of the hidden nature of mechanical engineering of mine rock, all kinds of geological disasters of mine, such as water inrush on the roof and floor, roof pressure, etc., are obviously nonlinear^13–15^. Therefore, the temporal and spatial evolution of internal stress in overlying strata of coal seam has typical nonlinear characteristics^16^.

Traditional mechanical methods face challenges in elucidating the nonlinear mechanics governing the evolution of mining-induced rock failure due to the inherent fuzziness and uncertainty within rock structures^17,18^. With the development of nonlinear dynamics theory, nonlinear dynamics of rock has become an important branch in the field of rock engineering. These studies are aimed at understanding the complex behavior of rock under the comprehensive action of multiple factors. In recent years, researchers have made remarkable progress in the development of nonlinear dynamic models. Aiming at the nonlinear phenomena in rock mining failure, various mathematical models dedicated to more accurately describing the nonlinear behavior of rock structure, fracture, slide and deformation have been proposed and applied, such as fractal theory^19,20^, chaos theory^21,22^, nonlinear time series analysis^23,24^. Due to the high nonlinearity and complexity of underground mining engineering, a large number of unstable and non-uniform mining stresses exist in the mining rock system, and there is material and energy exchange with the external environment. Nonlinearity is the essential characteristic of rock mining failure mechanical behavior. The nonlinearity of rock is manifested in the development of rock failure, deformation and stress from disorder to order, and the development of isokinetic stage to nonlinear.

Underground coal seam mining disrupts the initial stress equilibrium within the overlying rock, leading to significant alterations in the structure and mechanical properties of the rock. When rock reaches a new equilibrium state, its stress, deformation and energy will change accordingly, which will induce rock deformation, fracture and failure, and even mine dynamic disasters such as the relative movement of rock strata and the surface. This is a rather complex mechanical process, involving the overlying rock mechanical properties, geological structure conditions, mining length and mining speed and other factors. At the same time, the structural plane network composed of the primary fracture and the secondary fracture formed by mining has fractal characteristics^25–27^. However, in this complicated mechanical process, it can be found that the fracture failure of the goaf rock also has the regularity of fractal geometry. At present, the failure of mining rock is mainly concerned with the failure height of overlying rock^28–30^ and failure depth of floor^31,32^, on the other hand, there is a lack of in-depth research on the spatial–temporal evolution dynamic characteristics and spatial characteristics of the internal stress field of mining failure, which is more important for rock failure. Traditional rock stress evolution studies can only obtain the general law of stress regional distribution^31^. In fact, the change of mining rock stress has not only the characteristics of time series but also the characteristics of space series. However, the current research mainly focuses on the change of rock stress with mining distance^28–32^, and the relevant researchs on the change of rock stress from time dimension and space dimension are quite limited. In particular, the temporal and spatial characteristics of stress evolution of mining rocks are still unknown.

Based on the entropy and spatial–temporal cube coupling model, a time series model of mining overlying rock system is constructed by using nonlinear dynamics theory. Taking the Panel 11050 of Quandian Coal Mine in Henan Province, China as a geological prototype, the nonlinear stress evolution of overlying rock during mining is monitored by similar model tests. The spatial and temporal data of stress field of mining fractured rock are processed based on GIS spatial and temporal data analysis method, and the spatial and temporal cubic visual analysis model of stress field of mining fractured rock is established to reveal the spatial and temporal evolution of stress of mining fractured rock.

## Materials and methods

### Experimental materials

Although field tests, numerical simulations and similar material models during mining can all show the nonlinear stress evolution of the internal stress of rock. However, the field test is affected by many factors and the test period is long, and the numerical simulation needs appropriate parameters and boundary conditions, which is easy to differ greatly from the measured results. Relatively speaking, the similar material model has strong theoretical significance, so this paper adopts the similar material model to monitor the nonlinear stress evolution of rock stress. Similar materials model testing, rooted in the theory of similitude, serves as a method to investigate complex issues by leveraging similarities and analogies among various objects and phenomena. A similar materials model of coal seam roof is made according to the similar principle in the laboratory. Observing the mechanical parameters and their distribution within the model aids in deducing potential mechanical phenomena and the pressure distribution of the rock in the prototype, consequently addressing practical challenges in rock engineering production.

**Figure 1:**
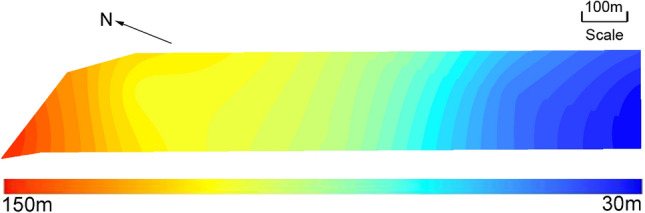
The specific thickness distribution of overburden.

The similar materials model is established based on the geological prototype of Panel 11050 in the Quandian Coal Mine. The mining thickness of Panel 11050 varies from 5 to 12 m, with an average thickness is 8 m. The dip angle of the coal seam ranges between 10° and 37°. The borehole reveals that the elevation of the Neogene basement ranges from −605 to −187.66 m, and the elevation of the coal seam floor ranges between −564.76 and −273.45 m. The thickness of the overburden in Panel 11050 is 30–150 m, and the specific thickness distribution is shown in Fig. 1. On the whole, the ground in this area is flat and the topography is not very undulating, and the bedrock surface is high in the north and low in the south. The hydrogeological and engineering geological conditions of the panel are complicated. Therefore, the panel can better reflect the stress entropy evolution characteristics of rock.

The test bench for the plane stress model has a size of length × width × height equal to 300 cm × 30 cm × 200 cm. According to the similarity theorem, the geometric similarity ratio *C*_L_ = 200, the time ratio *C*_t_ = 14.1, and the bulk density ratio *C*_γ_ = 1.67. The specific calculation is as follows^31^:

1 $$\left\{ \begin{gathered} C_{L} = \frac{{L_{1} }}{{L_{2} }} = \frac{200}{1} = 200 \hfill \\ C_{t} = \sqrt {C_{L} } = \sqrt {200} :1 = 14.1 \hfill \\ C_{\gamma } = \frac{{\gamma_{1} }}{{\gamma_{2} }} = \frac{2.5}{{1.5}} = 1.67 \hfill \\ \end{gathered} \right.$$

where, *L*_1_ is the prototype size, *L*_2_ is the model size, *γ*_1_ is the engineering prototype materials density, and *γ*_2_ is the similar materials density.

In the similar materials model, the coal seam is mined 200 cm, and 50 cm coal pillars are left on both sides. Sand is the aggregate for the similar materials and the cementing materials are chosen as the Kaolin, Gypsum and Calcium Carbonate. Coal seams in similar materials model are replaced by small wooden strips. Before the scale model test, the uniaxial compressive strength of specimens for the similar material with different ratios need to be tested to determine the reasonable ratio. The results are listed in Table 1.

**Table 1:** Key parameters of actual geological prototype and scaled materials components of similar materials.

Rock type	Lithology	Thickness (m)	Uniaxial compressive strength (MPa)	Material ratio				
				Sand (kg)	Kaolin (kg)	Calcium carbonate (kg)	Gypsum (kg)	Water (kg)
11	Sandstone	13.0	30.0	59.23	9.87	–	9.87	8.78
10	Mudstone interbedded with sandstone	34.0	10.0	180.73	18.07	–	7.75	22.95
9	Mudstone	16.0	8.0	85.05	8.51	–	3.65	10.80
8	Sandstone interbedded with mudstone	16.0	20.0	85.05	6.08	–	6.08	10.80
7	Sandy mudstone	7.6	12.0	40.40	4.04	–	1.73	5.13
6	Sandstone	9.4	35.0	48.95	2.45	–	5.71	6.35
5	Mudstone interbedded with sandstone	24.0	9.0	127.58	12.76	–	5.47	16.20
4	Sandstone	6.0	30.0	27.34	4.56	–	4.56	4.05
3	Mudstone	5.4	6.5	28.70	1.44	1.44	1.23	3.65
2	Sandstone	5.0	27.5	24.30	3.04	–	3.04	3.38
1	Sand-mudstone	10.0	15.0	53.16	5.32	–	2.28	6.75

### Methods

#### Spatial and temporal method

The overburden stress due to mining has both the spatial sequences and time series characteristics which affected by the spatial position and mining distance. Therefore, it needs to be analyzed simultaneously not only in time but also in space.

**Figure 2:**
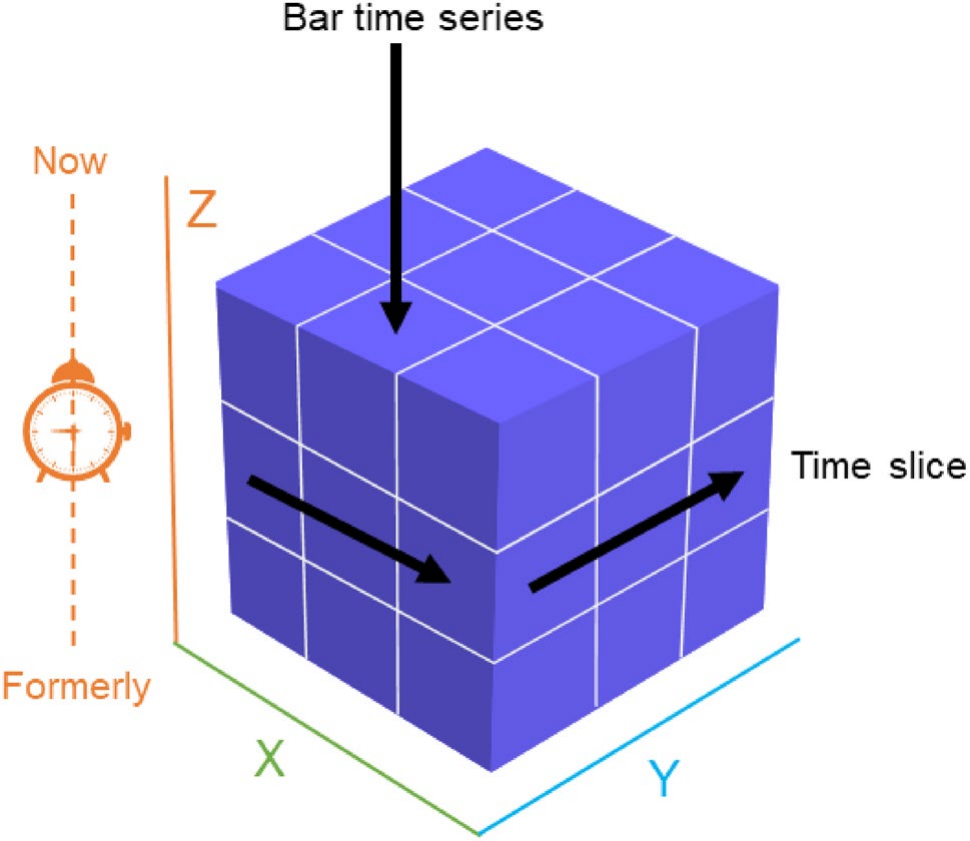
Spatial–temporal cube construction^35^.

The visualized analysis on the spatial–temporal is defined as the graphic of the spatial–temporal data which contain time, space and attribute information, to analyze their correlation or mining new data. The simple combination of “time” and “space” cannot represent spatial–temporal data due to the complicated relationship between each other. Hagerstand first proposed a spatial–temporal cube model, which was further studied by Rcker and Szego et al.^33,34^. The spatial–temporal cube model can be composed of a planar two-dimensional space and a one-dimensional time series as shown in Fig. 2. It can also be represented by a two-dimensional time series and a one-dimensional geospatial space, such as a two-dimensional time series characterized by “day” and “hour” intervals, respectively. A small cube in Fig. 2 can represent a geographical phenomenon at a fixed day and a fixed hour point, while the entire cube can represent the process of the geographical phenomenon at different day and different hour points.

**Figure 3:**
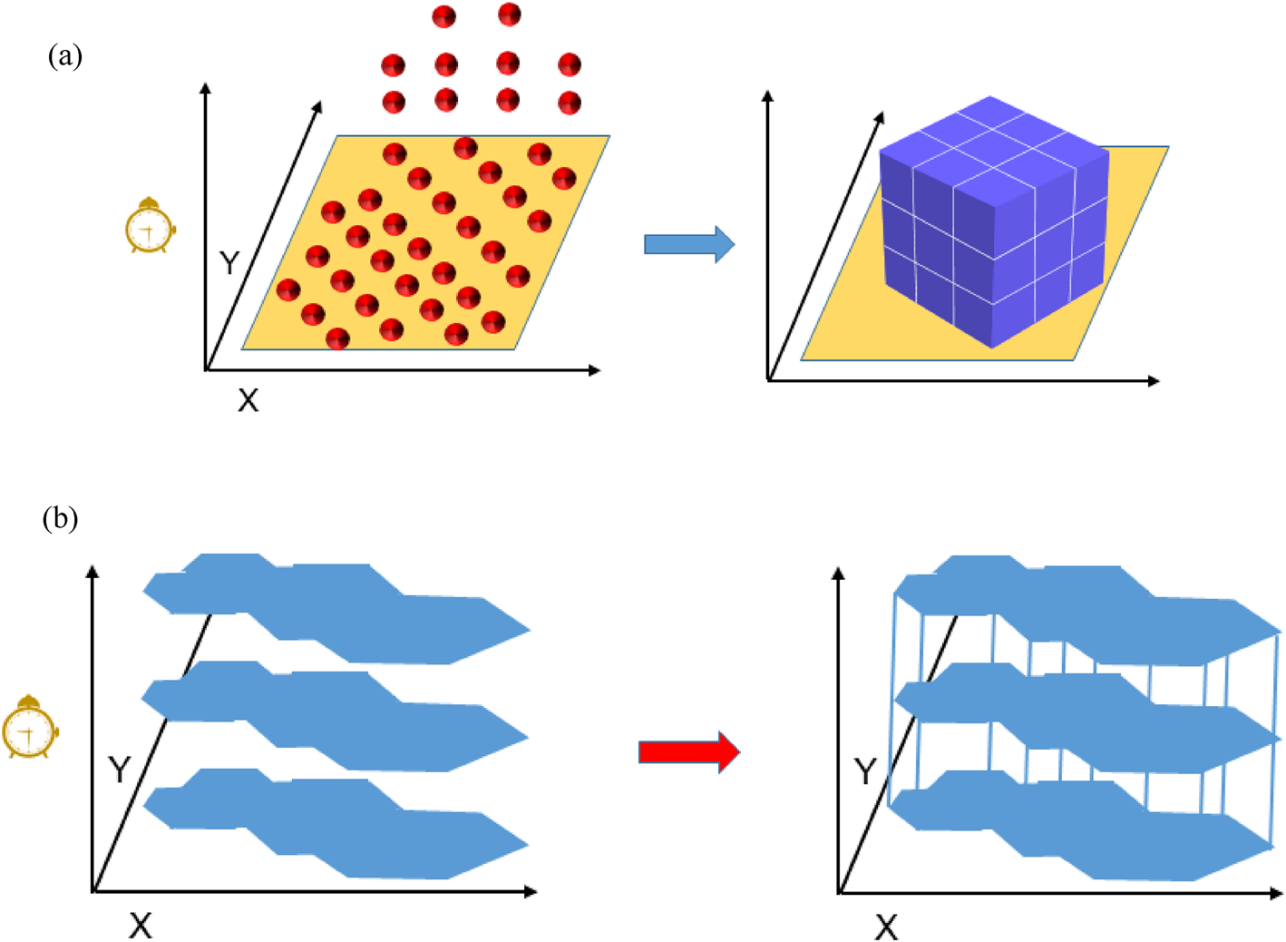
The process of spatial–temporal cube^35^. (**a**) Spatial–temporal cube model based on the aggregation points, (**b**) spatial–temporal cube model based on the defined location.

As shown in Fig. 3, the spatial–temporal cube model can be built in two ways. The first is to build a spatial–temporal cube model through aggregation points (Fig. 3a). The second is to build a spatial–temporal cube model by predefined locations (Fig. 3b). Aggregate spatial and temporal bars into netCDF data structures to create a spatial–temporal cube model in which each bar contains a location, time, and one or more attribute values or variables.

This paper uses the spatial–temporal cube model to analyze the observed data of the overburden stress in the mining process. Due to the limitation of the monitoring points and the certainty of the location, the defined location is used to create a spatial–temporal cube model. The monitoring points in the same position will have a location ID (LOCATION_ID), and only the location of the stress data in the monitoring points will be analyzed in the spatial–temporal data processing.

#### Establishing stress entropy to predict stress catastrophe

The concept of the information entropy was firstly proposed by Shannon in 1948^36,37^.

2 $$S = - \sum\limits_{i = 1}^{n} {p_{i} } \log p_{i}$$

where *S* is the information entropy, *p*_i_ indicates the probability of the information. Information entropy reflects the disorder degree of the system information. The smaller information entropy results in the smaller disorder degree of the system which means the system is more orderly, and vice versa.

The stress entropy is proposed to characterize the spatial–temporal features of the stress in the overburden during the mining process. The definition of the information entropy is shown as Formula (3).

3 $$K_{\sigma } = - \sum\limits_{i = 1}^{n} {\frac{{\sigma_{i} }}{{\sum {_{i = 1}^{m} \sigma_{i} } }}} \ln \left( {\frac{{\sigma_{i} }}{{\sum {_{i = 1}^{m} \sigma_{i} } }}} \right)$$

where *K*_σ_ is the stress entropy, *σ*_i_ is the observed stress at a certain moment. The stress entropy is influenced by the mining progress and time, which can reflect the influence of the mining rate on the stress. Furthermore, the stress entropy may change in advance or lag when the rock breaks, which can indicate the evolution state of the stress in the overburden system. The upward trend of stress entropy indicates that the order of overlying rock system decreases and the disorder increases due to coal seam mining.

#### Man-Kendall trend analysis

Man-Kendall trend analysis method, as an effective method of spatial–temporal statistical analysis, is often used to test the trend of time series. Define a time series *Y* and assume the number of samples is *n*, then the null hypothesis and alternative hypothesis in Man-Kendall are as follows. Null hypothesis A: time series *Y* is an independent sample with random probability and the same distribution; alternative hypothesis B: for any *i*, *j* ≤ *n*, and *i* ≠ *j*, *Y*_i_ and *Y*_j_ are distributed differently. Construct the test statistic *T*^35^:

4 $$\left\{ {\begin{array}{*{20}c} {T_{{\text{n}}} { = }\sum\limits_{{\text{i = 1}}}^{{\text{n - 1}}} {\sum\limits_{{\text{j = i + 1}}}^{{\text{n}}} {{\text{Sgn(Y}}_{{\text{j}}} {\text{ - Y}}_{{\text{i}}} {)}} } } \\ {{\text{Sgn(Y}}_{{\text{j}}} {\text{ - Y}}_{{\text{i}}} {\text{) = 1,Y}}_{{\text{j}}} {\text{ > Y}}_{{\text{i}}} } \\ {{\text{Sgn(Y}}_{{\text{j}}} {\text{ - Y}}_{{\text{i}}} {\text{) = 0,Y}}_{{\text{j}}} {\text{ = Y}}_{{\text{i}}} } \\ {{\text{Sgn(Y}}_{{\text{j}}} {\text{ - Y}}_{{\text{i}}} {\text{) = - 1,Y}}_{{\text{j}}} {\text{ < Y}}_{{\text{i}}} } \\ \end{array} } \right.$$

For *n* ≥ 10, the statistic *T* approximately follows a normal distribution, and we can see that its mean is 0 and its variance is:

5 $$s^{2} = \frac{n(n - 1)(2n + 5)}{{18}}$$

Its standardized test statistic *z* is:

6 $$z = \left\{ {\begin{array}{*{20}c} {(T - 1)/\left| s \right|,T > 0} \\ {0,T = 0} \\ {(T + 1)/\left| s \right|,T < 0} \\ \end{array} } \right.$$

For the trend test, assuming the significance level is *α*, then the confidence *p*=1-*α* is true for the following:

7 $$|z| > z_{\alpha /2}$$

If the original hypothesis is excluded, it can be seen that the sequence has obvious changing trend. Therefore, the z-score is obtained in the standardized test statistics, and the trend characteristics of the sequence can be judged by the z-score. When it is greater than 0, the higher the value is, the more obvious the ascending trend of the sequence; when it is less than 0, the smaller the value is, the more obvious the descending trend of the sequence.

## Experimental process

Data collection is performed using a soil pressure sensor. The thickness of the loose layer of the Neogene and the Quaternary is relatively large. In the scale model test, the external load is used to simulate the loose layer load with a loading time of more than 24 h. The load is calculated as follows.

8 $${\text{q}}_{{\text{m}}} = \frac{{\gamma_{{\text{a}}} (H - H_{{\text{m}}} )}}{{C_{\gamma } C_{l} }} \cdot l \cdot b$$

where, *H* is the burial depth of the coal seam, m; *H*_m_ is the thickness of the simulated overburden, m; *γ*_*a*_ is the average bulk density, kN/m^3^; *l*, *b* are the length and width of the scale model, respectively, m. And then the external load is calculated as *q*_m_ = 31.70 kN.

**Figure 4:**
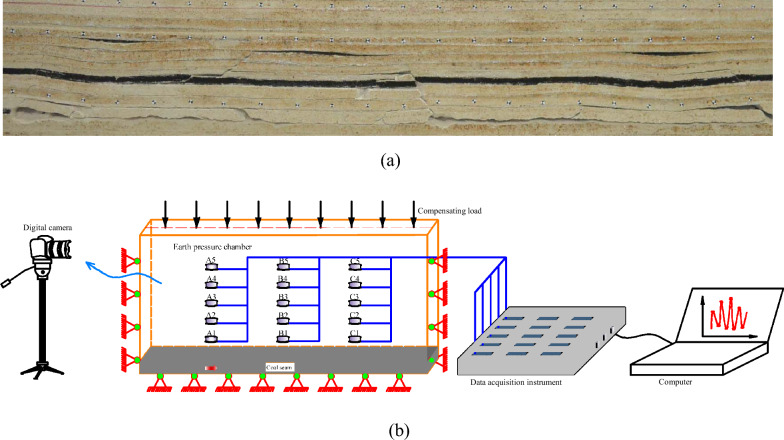
Layout of sensors. (**a**) Actual pictures of the model after excavation. (**b**) Schematic diagram of the monitoring arrangement.

3 monitoring lines with 5 monitoring points on each line are set up in the horizontal direction, which are used to monitor the evolution characteristics of stress and strain in the mining-induced overburden. Moreover, the stress sensors are arranged in the model with an array of 5 rows and 3 columns. The details are shown in Fig. 4. The distance between the three columns of monitoring lines and the open-off cut was 25, 100 and 175 cm, respectively. The distance between the five rows of monitoring lines and the coal seam floor is 11.5, 23.2, 33.9, 47.7 and 61.7 cm, respectively.

The stress evolution is observed using the resistance miniature soil pressure sensors with the XY-TY02A type, and the corresponding data acquisition is implemented using DT85 Data-taker with an intelligent program. The DT85 Date-taker is an independent adaptive system with a controllable 24 V power supply, which can support SDI-12 sensors networking, Modbus and FTP and Web interfaces of SCADA system. The inputs of the DT85 include analog and digital quantities, high-speed counters, impulse input, programmable sensor serial channels, and CANgate interfaces for CANbus.

The mining speed is simulated according to the time ratio based on the actual advancing speed. That is, each mining step is 10 cm means the prototype of 20 m, the mining thickness is 4 cm means the prototype is 8 m, the mining length is 200 cm means the prototype is 400 m. Each time the mine is mined, the small wooden strips representing the coal seam in the similar material model are manually removed by 10 cm. To investigate the time effect of stress in the mining-induced overburden, the scale model is mined every 17.5 h according to the actual mining schedule of 2 m per day. Each monitoring point is initialized into the same value and collected every 0.5 h with a frequency of 1 HZ for 1 min at each acquisition time automatically. The model is implemented within 15 days.

The variation law of stress in the mining-induced overburden with mining distance is a comprehensive manifestation of the progressive instability process of overburden system. Based on the time effect of mining overburden failure, the entropy change and the spatial–temporal evolution of overburden stress during mining process are investigated according to the time dimension.

## Results

**Figure 5:**
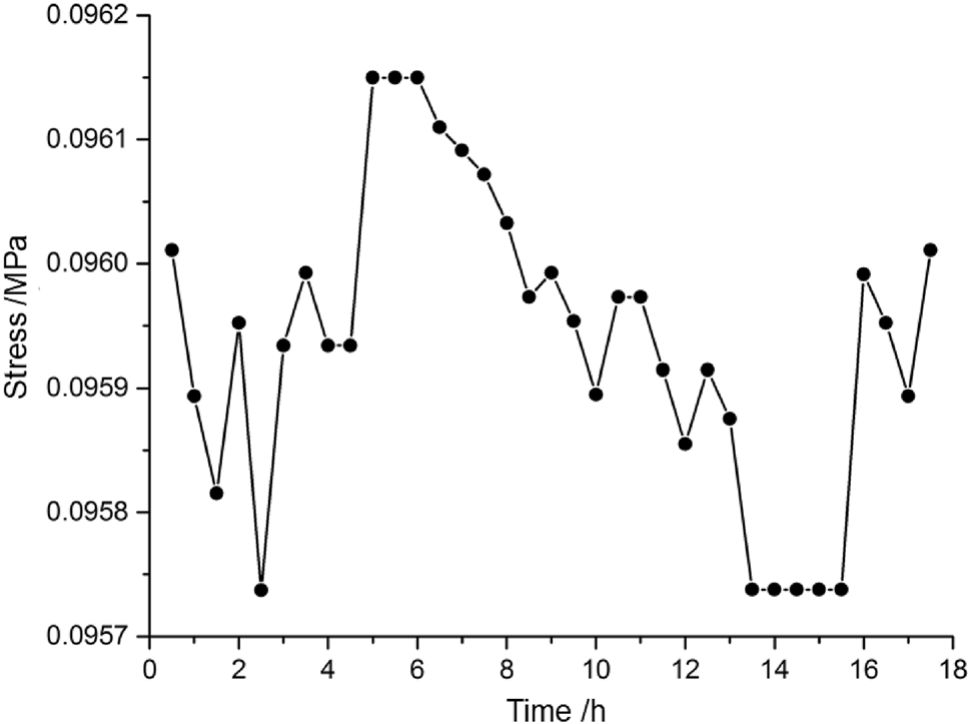
Stress change of A1 point during 10 cm mining.

Each mining step has a certain period of time interval in the model mining process, and then the data acquisition is processed during this interval. The vertical stress may gradually rebalance which is important to the mining safety. As shown in Fig. 5, the monitoring point of NO. A1 has a stress entropy of 3.55 during the first mining time period with a mining distance of 10 cm in the scale model or 20 m in the actual panel.

**Figure 6:**
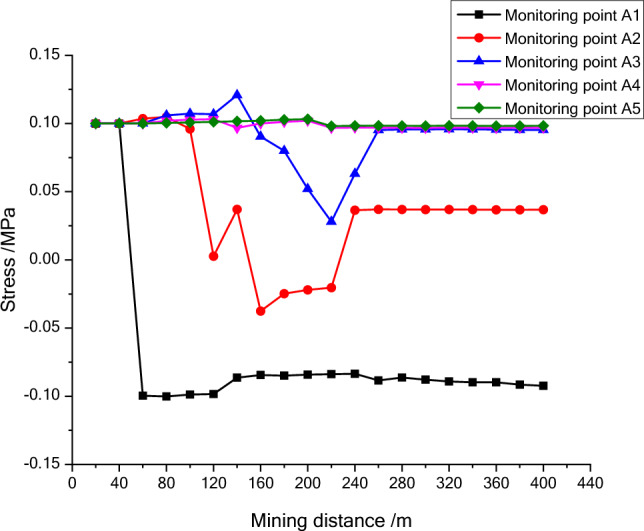
The stress variation map of the first line monitoring points during mining process.

**Figure 7:**
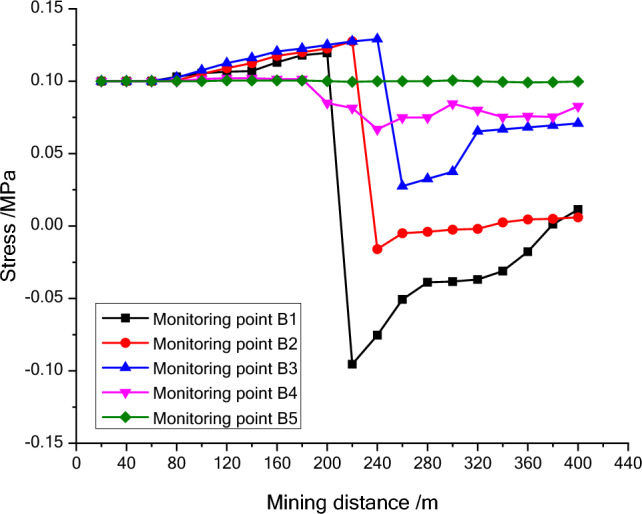
The stress variation map of the second line monitoring points during mining process.

**Figure 8:**
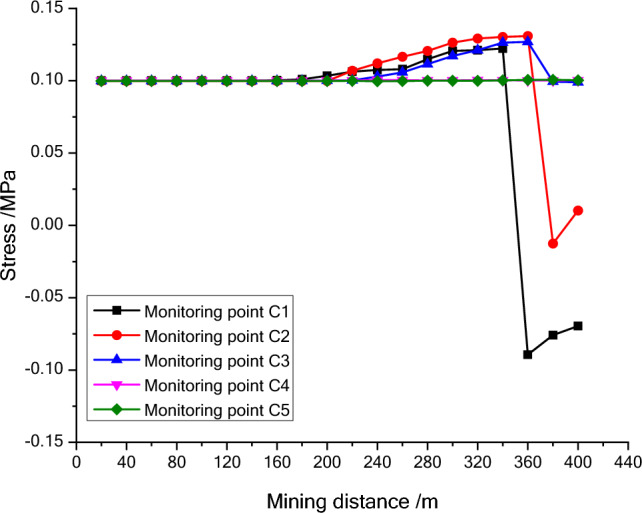
The stress variation map of the third line monitoring points during mining process.

**Figure 9:**
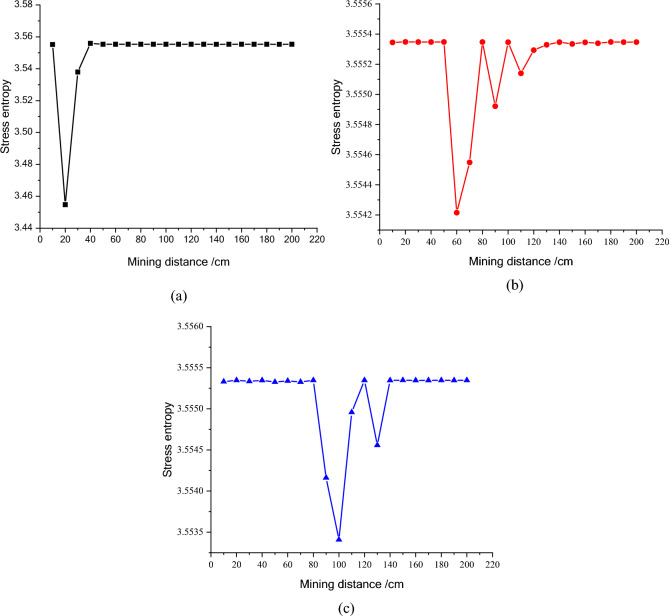
The entropy of stress of the first line monitoring points during mining process. (**a**) NO. A1, (**b**) NO. A2, (**c**) NO. A3.

**Figure 10:**
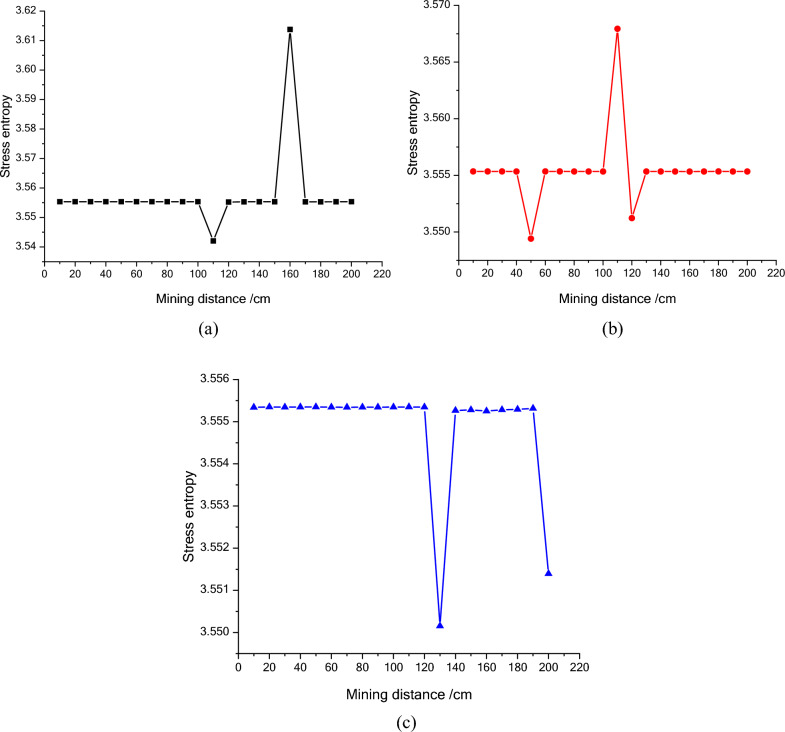
The entropy of stress of the second line measuring points during mining process. (**a**) NO. B1, (**b**) NO. B2, (**c**) NO. B3.

**Figure 11:**
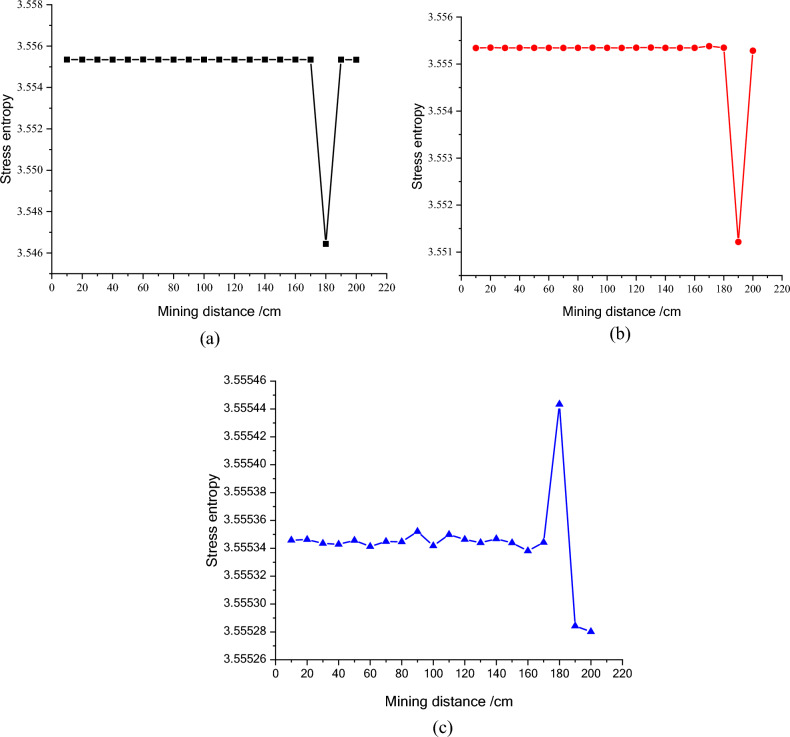
The entropy of stress of the third line monitoring points during mining process. (**a**) NO. C1, (**b**) NO. C2, (**c**) NO. C3.

As shown in Figs. 6, 7 and 8, the stress of the measurement point above 47.7 cm from the bottom of the coal seam changes slightly and the same to its entropy. And then, the stress entropy of other monitoring points is calculated with the time series and shown in Figs. 9, 10 and 11. The model keeps in a stable state at the initial stage of mining. However, a small change may come out with the mining process and form a goaf in the panel.

The stress entropy mentioned in this paper is mainly concerned with the location of abrupt change points. The upward trend of stress entropy indicates that mining increases the disorder of overlying rock system, while the downward trend of stress entropy indicates that mining increases the orderliness of overlying rock system. When the mining distance processed between 40 and 60 m, the measuring point of NO. A1 at 23 m (model 11.5 cm) above the goaf is relieved, and the stress entropy changes in advance, while the stress entropy of the point A2 changes lag, which indicate that the stress entropy will abruptly change in advance when the stress suddenly decrease and the entropy changes lag when the stress increases. The stress of point A2 has been increasing resulting in the energy increase in the overburden. Once the energy exceeds the rock ultimate strength, the overburden will break and cave, and finally result in the stress release at this position. From the mining distance of 120 m, the stress of the A1 measuring point gradually recovers. The change of the stress in the second column during the mining process reflects the variation of the stress during the mining process of the panel. And the unmined rock in a certain range in front of the panel has a stress rising zone and gradually changes along with the mining process. However, the stress in the lower rock layer of the goaf is small. Furthermore, the vertical stress in the overburden in front of the panel changes periodically with the mining process, and each stress mutation is accompanied by a sudden stress entropy change.

The change of stress entropy in the three monitoring lines is consistent with the change of stress, in that the stress in column A is affected by rock mining first, then in column B, and finally in column C. As can be seen from Figs. 9, 10 and 11, the stress entropy of rocks closer to the coal seam is larger, while that of rocks farther away from the coal seam is smaller. This shows that the closer to the coal seam, the greater the influence of rock mining on the rocks, and the stronger the disorder of the overlying rock system in the corresponding area. The energy will be accumulated in the overburden due to the increase in stress before the rock strata is failure. When the overlying strata is damaged, the accumulated energy in the overburden will be dissipated and leading to the unload of the overburden. And the stress in the goaf cannot recover to the initial stress value. However, a high stress may highlight in the un-mined coal body and the rock layer above a certain height in front of the panel. It fully proves that there is a dynamic stress arch in the surrounding rock of the stope, which mainly bears and transmits the load and pressure of the overlying rock.

## Discussion

The temporal and spatial visual analysis of the variation of overburden stress can be performed based on its diachronic nature. Table 2 lists the data of the monitoring point A1 at 0:00 and 17:30 on January 19, 2018, wherein the stress value is the average value in the time period.

**Table 2:** Average stress in overburden (first 35 h).

Monitoring point	Date (month/day/year)	Time	Stress (MPa)	Date (month/day/year)	Time	Stress (MPa)
A1	01/19/2018	0:00	0.0999	01/19/2018	17:30	0.099900
A2	01/19/2018	0:00	0.1000	01/19/2018	17:30	0.100000
A3	01/19/2018	0:00	0.1000	01/19/2018	17:30	0.100000
B1	01/19/2018	0:00	0.1000	01/19/2018	17:30	0.100036
B2	01/19/2018	0:00	0.1000	01/19/2018	17:30	0.100000
B3	01/19/2018	0:00	0.1000	01/19/2018	17:30	0.100000
C1	01/19/2018	0:00	0.1000	01/19/2018	17:30	0.100000
C2	01/19/2018	0:00	0.0998	01/19/2018	17:30	0.099763
C3	01/19/2018	0:00	0.0999	01/19/2018	17:30	0.099917

**Figure 12:**
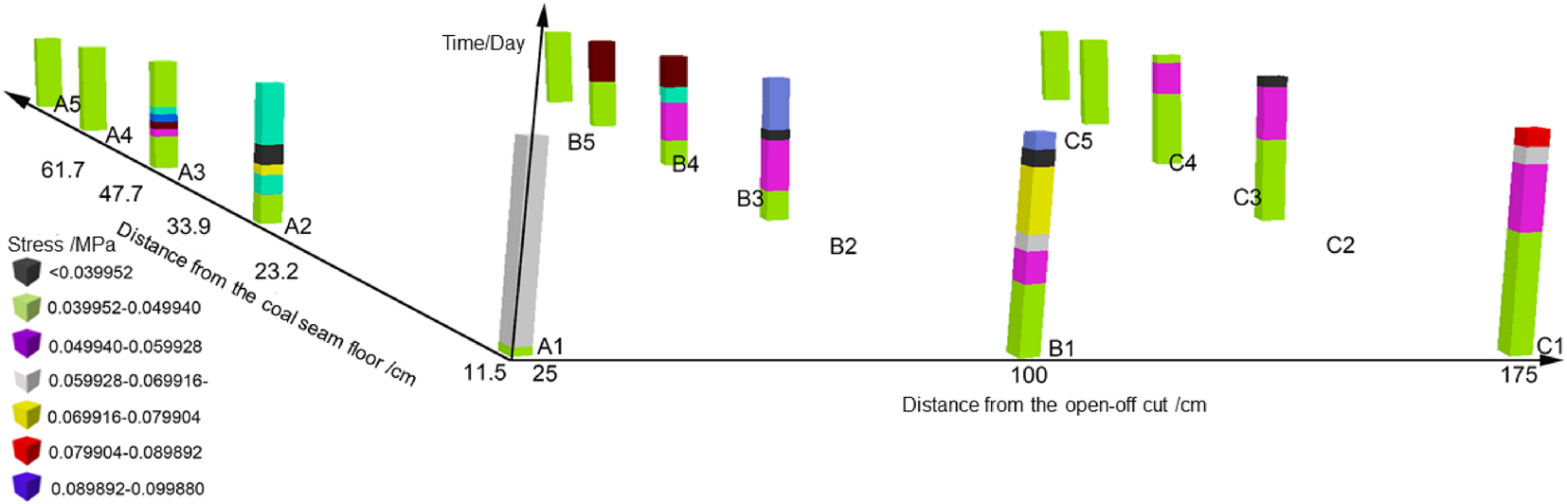
Spatial–temporal cube model of stress of overburden due to mining.

Based on GIS, the spatial–temporal cube model of stress in the mining-induced overburden is established by taking the position of the monitoring point as the planar two-dimensional coordinate, and the time *T* as the third dimension coordinate. The model is displayed in a 3D scene and the result is processed, as shown in Fig. 12. The Man-Kendall trend analysis is carried out on the variation of stress over time. The z-score is calculated as -1.86 according to the 4200 values in a total of 300 observations, which indicates a significant reduction in the overall stress in the overburden over time.

**Figure 13:**
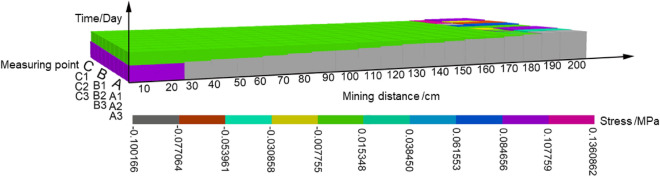
Spatial–temporal cube of stress of overburden due to mining.

A three-dimensional coordinates contain the mining distance, the monitoring time and the different monitoring points, is used to analyze the spatial–temporal evolution of the overburden stress in the time period of each mining step during the mining process. The stress values are represented by different colors considering the monitoring points in and around the water-conducting fractured zone. Figure 13 shows the spatial–temporal evolution characteristics of overburden stress in the equilibrium period of each mining step. The Man-Kendall trend analysis is also carried out on the variation of stress over time. The z-score is calculated as -2.63 according to the 2520 estimated values in 180 observations. Results indicate that the overburden stress in the water-conducting fractured zone has a significant decrease over time, and the lower trend is more obvious than that of the overall stress in the panel. However, the traditional rock stress evolution study with similar models can only get the stress change of a single point, but not the stress trend evolution of the whole region^31^. It can be seen that the rock stress model based on the combination of entropy and a spatial–temporal cube can better predict the spatial–temporal evolution of rock stress, and better predict the overall failure of rock system. In the future research work, the mine pressure in the mining process should be analyzed, and the spatial–temporal cube model of mine pressure evolution should be established according to the observed data. The spatial–temporal visualization analysis and hot spot analysis of the spatial–temporal evolution characteristics of mine pressure are carried out to reveal its disaster-inducing mechanism, and the mine disaster caused by corresponding stress is predicted.

## Conclusions

This paper aims to develop a spatial–temporal visualization model for analyzing the stress in overburden based on GIS and the information theory. The stress entropy is proposed to analyze the spatial–temporal evolution characteristics of overburden stress due to mining. The main findings are summarized as follows:

1. The engineering geological model is established based on Panel 11050 of Quandian Coal Mine. Through the similar material model test, the stress in the fractured overburden is monitored during the mining process. Then the diachronic characteristics and entropy of the overburden stress during the mining process are analyzed based on information theory and the spatial–temporal model from the perspective of system science.
2. Based on GIS spatial–temporal visualization model, the time series and spatial sequence of overburden stress during mining process are analyzed, and the spatial–temporal evolution of the overburden stress is visualized. The vertical stress in the overburden in front of the panel changes periodically with the mining process, and each stress mutation is accompanied by a sudden stress entropy change.
3. The spatial–temporal cube model is established to visualize spatial–temporal evolution of the overburden stress, and the trend analysis is carried out on the spatial–temporal model for the mining-induced overburden stress. The z-score is calculated as −1.86 which indicates a significant reduction in the overall stress in the overburden over time. The spatial–temporal cube model of the overlying rock stress in the water-conducting fractured zone has a z-score of −2.63, which indicates that the decrease effect of the overlying strata stress is much more significant over time.

## Data Availability

The datasets used and/or analysed during the current study available from the corresponding author on reasonable request.
